# Mania Without a Past: A Case of Discontinuation-Induced Mania in a Non-Bipolar Migraine Patient

**DOI:** 10.1192/j.eurpsy.2026.11203

**Published:** 2026-07-31

**Authors:** F. Karsavurdan, O. Metin, A. Erdogan

**Affiliations:** Psychiatry, Akdeniz University Hospital, Antalya, Türkiye

## Abstract

**Introduction:**

Antidepressant discontinuation, dose reduction, or abrupt cessation after a period of use may lead to a well-recognized clinical condition known as Antidepressant Withdrawal Syndrome. This syndrome is characterized by symptoms such as anxiety, “electric shock-like” sensations, nausea, and dizziness. However, beyond these common manifestations, there exists a rare, paradoxical, and less well-known clinical phenomenon associated with rapid dose reduction or abrupt discontinuation of antidepressants, termed Antidepressant Discontinuation Mania. Cases have been reported across nearly all classes of antidepressants. Diagnostic criteria for this condition were first proposed by Haddad & Anderson (Adv Psychiatr Treat 2007;13:447-5): Criteria for antidepressant discontinuation mania include: (1) Onset of mania following antidepressant cessation or dose reduction; (2) Absence of alternative pharmacological explanations (e.g., stimulant use, withdrawal of mood stabilizers or antipsychotics); (3) At least four weeks of prior continuous antidepressant use; (4) Symptom onset within one week of discontinuation or dose reduction.

**Objectives:**

To present a rare case of antidepressant discontinuation mania and highlight the clinical importance of recognizing this underreported phenomenon.

**Methods:**

Clinical case report and brief literature review

**Results:**

A 38-year-old female patient with no personal or family history of bipolar disorder had been using venlafaxine 225 mg/day for one year as migraine prophylaxis. The Neurology Department rapidly reduced the dose to 75 mg/day over 8 days. On the eighth day, the patient developed restlessness, significant insomnia, impulsive spending, and distractibility.

By day 14, psychiatric evaluation revealed elevated mood, mild psychomotor agitation, decreased need for sleep, increased thought flow, and reduced attention span. No psychotic symptoms or suicidal ideation were observed. A clinical diagnosis of discontinuation mania was made.

Treatment was adjusted by increasing venlafaxine to 150 mg/day and initiating quetiapine XR 300 mg/day plus 25 mg IR at night. Full remission was achieved within one month, with functional recovery observed.

Two years later, venlafaxine was gradually discontinued without relapse.

**Conclusions:**

Despite various hypotheses—such as latent bipolarity—there is no scientific consensus on the etiology of antidepressant discontinuation mania. Misdiagnosis as primary bipolar disorder may lead to unnecessary long-term mood stabilizer use, highlighting the need for clinical awareness. Our case, featuring mania without prior mood disorder, differs from most reports and may aid in elucidating both antidepressant neurobiology and bipolar pathophysiology.

**Disclosure of Interest:**

None Declared

